# Commentary: Selective Development of Anticorrelated Networks in the Intrinsic Functional Organization of the Human Brain

**DOI:** 10.3389/fnhum.2017.00013

**Published:** 2017-01-23

**Authors:** Marie Arsalidou, Maksim G. Sharaev, Tatyana Kotova, Olga Martynova

**Affiliations:** ^1^Department of Psychology, National Research University Higher School of EconomicsMoscow, Russia; ^2^Department of Psychology, York UniversityToronto, Canada; ^3^National Research Centre “Kurchatov Institute”Moscow, Russia; ^4^Cognitive Research Lab, Russian Academy for National Economy and Public Administration (RANEPA)Moscow, Russia; ^5^Centre for Cognition and Decision Making, National Research University Higher School of EconomicsMoscow, Russia

**Keywords:** development, default-mode network, executive attention, functional connectivity, resting state, fMRI

As adults we solve problems by applying our executive know-how and directing our mental-attention to relevant information. When we are not problem solving, our mind is free to wonder to things like lunchtime; this is often referred to as the default-mode. It is established that for adults the relation among executive and default-mode brain areas is negative (Fox et al., 2005; Arsalidou et al., 2013). Parts of the prefrontal cortex are involved in both the executive and default-mode networks.

Chai et al. (2014) examined the relation among executive and default-mode areas in children, adolescents and adults, using resting-state fMRI. This is likely the most thorough, methodologically sound paper to date that investigates this relation across typical development. Although it was unclear whether data adhered to assumptions for parametric tests such as homoscedasticity, as Pearson's correlations are sensitive to outlier data, the authors examine different aspects of their data by (a) controlling for movement and artifact, (b) performing group analyses and covarying outliers, and (c) repeating the analyses using global signal regression. Overall, results show that across groups (8–12, 13–17, and 18–24 year-olds), relations among executive and default-mode areas are heterogeneous. In many regions the relation among executive and default-mode areas are positive in children. For adolescents it becomes more anti-correlated (i.e., more adult-like), eventually converting to a negative relation in adults. The authors highlight that functional connectivity reversal cannot be accounted by performance differences *per se*, because functional images were recorded at rest.

Results are fascinating and understandably interpreting such findings is challenging. The authors suggest that lower performance on working memory and cognitive control generally found in younger children may relate to the lack of anti-correlation. Yet they report that Intelligence Quotient (IQ) scores did not correlate with age, suggesting that participants were cognitively comparable. Why is this relation positive in children? We believe what is lacking in Chai et al. (2014) is a developmental theory-based interpretation. Here we highlight that the mechanisms that underlie this change from positive correlation in children to negative correlation in adults may be driven not only by cognitive processes but also by affective processes that develop across those formative years.

We define affective processes within the developmental theory of constructive operators (Pascual-Leone and Johnson, 2005; Pascual-Leone, 2012). Although emotions and cognitions can assign vital and truth value to a situation, respectively, pure affects have innate, evolutionary foundations. Specifically, unlike pure affects, emotions have to cross boundaries with cognition in order to apply and be perceived (Pascual-Leone et al., 2015; Arsalidou and Pascual-Leone, 2016). Younger children are more likely to be drawn by the here-and-now so that salient features of a situation are more likely to motivate their decisions.

Consider the famous marshmallow experiment, when 4-year-olds were asked to either eat one marshmallow in their plate or wait and get a second marshmallow when the experimenter returned (Mischel et al., 1972). The majority of children did not wait and ate the one delicious marshmallow, whose salience discounted the value of two marshmallows in the future. According to Bar (2010), future-oriented processes elicit activity in areas such as the ventromedial prefrontal cortex. This region is also critical for processing all sorts of rewards (Sescousse et al., 2013) and the default-mode network (Spreng et al., 2009). Notably, anterior default-mode regions show more heterogeneity, in their anatomical and functional connections, rather than posterior and ventral default-mode regions (Sharaev et al., 2016), perhaps suggestive of their dynamic role in the formation of prefrontal subsystems, as we know them in adults.

Personal wants, needs, feelings, and emotions may not clearly differentiate from cognitive decisions for children, giving rise to a positive relation between default-mode and executive brain areas. We need to consider the relational state among personal wants, current situation, and cognitive aptitude (i.e., mental-attentional capacity, inhibition). Since children are less likely to plan ahead in order to satisfy their personal wants they mainly focus on the current situation. Moreover, cognitive aptitude is inevitably linked to brain maturation. The prefrontal cortex, in particular, has a protracted development (Gogtay et al., 2004), which corresponds to a period of significant cognitive improvements (Arsalidou et al., 2010; Powell et al., 2014). According to developmental theory, children between 8 and 12 years have a mental-attentional capacity between 3 and 5 symbolic scheme-units (Pascual-Leone, 1970; Arsalidou, 2013). Adolescents, ages 13–17, however have a mental-attentional capacity between 6 and 7, a number similar to that of adults (Miller, 1956, Pascual-Leone, 1970). As the amount of information children can effectively hold and manipulate in mind increases the more able they become to control personal processes and more able to direct their thoughts to future-oriented processes (Pascual-Leone, 2014). Until higher-order executive schemes for planning and inhibition develop, areas related to default-mode and executive networks would activate together. Anticorrelations observed in adolescents and adults are likely a consequence of a learned functional pattern or coping strategy that helps the person maintain balance between current reality and stimulus-independent thoughts.

Work by Chai et al. (2014) has important practical and theoretical implications. Practically future fMRI studies should not average over large age-ranges as critical differences are observed between children and adolescents. Moreover, given the intricate relation between cognitive and affective processes across development, future connectivity studies may consider administering measures that evaluate core cognitive abilities such as mental-attentional capacity (e.g., Arsalidou and Im-Bolter, 2016) and measures that evaluate affective development such as theory of mind (e.g., Happé, 1994), perspective taking (e.g., Dixon and Moore, 1990), motivation (e.g., Lepper et al., 2005), or self-control (e.g., Achenbach, 1991). Theoretically, it would be fascinating to see at which age exactly these transitions occur in the brain and whether they adhere to developmental predictions. Although, adult task-based neuroimaging studies assessing meta-awareness (i.e., noting the current contents of consciousness) and mind-wandering have being done (Schooler et al., 2011), no such studies are available with children, perhaps because methods for addressing this questions are not yet available. Research in this direction would fuel the construction of improved developmental neurocognitive models.

## Author contributions

All authors listed, have made substantial, direct and intellectual contribution to the work, and approved it for publication.

## Funding

MA was supported by the Natural Sciences and Engineering Research Council (NSERC) of Canada. MS was supported by a junior grant from the Russian Foundation for Basic Research, RFBR Project 16-34-00558 mol_a, TK was supported by the Russian Foundation for the Humanities project No-15-06-10943, and OM was supported by the Russian Academic Excellence Project “5-100.”

### Conflict of interest statement

The authors declare that the research was conducted in the absence of any commercial or financial relationships that could be construed as a potential conflict of interest. The reviewer KR and handling Editor declared their shared affiliation, and the handling Editor states that the process nevertheless met the standards of a fair and objective review.
